# Gene Flow and Genetic Variation Explain Signatures of Selection across a Climate Gradient in Two Riparian Species

**DOI:** 10.3390/genes10080579

**Published:** 2019-07-31

**Authors:** Tara Hopley, Margaret Byrne

**Affiliations:** Biodiversity and Conservation Science, Department of Biodiversity, Conservation and Attractions, Locked Bag 104, Bentley Delivery Centre, WA 6983, Australia

**Keywords:** climate change, connectivity, environmental association, genomics, selection, SNP

## Abstract

Many species occur across environmental gradients and it is expected that these species will exhibit some signals of adaptation as heterogeneous environments and localized gene flow may facilitate local adaptation. While riparian zones can cross climate gradients, many of which are being impacted by climate change, they also create microclimates for the vegetation, reducing environmental heterogeneity. Species with differing distributions in these environments provide an opportunity to investigate the importance of genetic connectivity in influencing signals of adaptation over relatively short geographical distance. Association analysis with genomic data was used to compare signals of selection to climate variables in two species that have differing distributions along a river traversing a climate gradient. Results demonstrate links between connectivity, standing genetic variation, and the development of signals of selection. In the restricted species, the combination of high gene flow in the middle and lower catchment and occurrence in a microclimate created along riverbanks likely mitigated the development of selection to most climatic variables. In contrast the more widely distributed species with low gene flow showed a stronger signal of selection. Together these results strengthen our knowledge of the drivers and scale of adaptation and reinforce the importance of connectivity across a landscape to maintain adaptive potential of plant species.

## 1. Introduction

Global climate change is predicted to lead to changes in local climate, particularly changes in temperature and precipitation [1]. Climate is a significant driver of the range of a species, and global patterns have shown significant range shifts across many species in response to changing climates [2,3,4,5]. Range shifts require habitat to be available, and this is currently challenging given the scale of land clearing that has occurred and the need for migration over time frames that are much shorter than they have been historically [4,6]. In addition to range shifts, the opportunities for species to respond to changing environments include adaptation to new environmental conditions, and/or response via plasticity in situ [7]. The range of a species usually encompasses at least some variation in environment or climate, with many species occurring across environmental gradients, and it is expected that these species will exhibit some level of adaptation as heterogeneous environments and localized gene flow will facilitate local adaptation [8]. This adaptation is likely to be advantageous in stable environments but may be disrupted by a changing climate [9], yet can be harnessed in climate adaptation strategies [10,11]. 

Ecological resilience of ecosystems under a changing climate is likely to encompass both adaptation and plasticity in species [12]. The patterns of adaptation and standing genetic variation within species are influenced by gene flow, which is impacted by distribution and connectivity of populations [13]. High genetic connectivity maintains high standing genetic diversity and the potential for future selection of adaptive genes, and under high gene flow, strong selection pressure is needed for local adaptation to develop [13,14]. Genetic connectivity will also facilitate shared phenotypic plasticity [15], although populations at the outer edges of a species range may already be at the limits of its ability to respond to environmental change. Species at range edges may have limited capacity to adapt or respond through plasticity due to lower effective population sizes, lower standing genetic variation and gene flow, and higher rates of genetic drift [16]. Understanding patterns of adaptation and plasticity and their interaction with gene flow, connectivity and range distribution is critical for development of climate adaptation strategies to manage ecosystems subject to changing climatic conditions.

Across environmental gradients, populations at the range edge currently adapted to warmer drier climates may harbor crucial genes for persistence under these conditions, and functional connectivity between these and central populations will be critical to allow for the migration of adapted genes as climate changes [17]. Habitat fragmentation can lead to increased isolation, reduced gene flow, and a reduction in genetic variation [18,19], all factors that have negative consequences for the ability of species to adapt and/or persist in changing environments. Mitigating the effects of habitat clearing and fragmentation will improve the response of species to impacts of changing climate. Where opportunities for natural genetic connectivity are low, assisted gene migration [11], and climate-adjusted provenancing as a seed sourcing approach for restoration [10] have been proposed as climate adaptation strategies for ecosystem management that harness the potential adaptive variation present in species.

Theory on local adaptation predicts that species with high gene flow will show less local adaptation, unless selection pressure is strong [12,20,21]. Studies of adaptation in tree species have found associations with climate variables, and have generally been undertaken on species that have high, long distance dispersal (i.e. wind pollination) and are geographically distributed over large spatial scales (over 500 km) [22,23,24,25,26]. A study over more moderate distances (100–500 km) by Pais et al. [27] on the insect pollinated Dogwood (*Cornus florida*), also found adaptation to climate variables in a species with high gene flow. Similar studies over moderate distances have shown signals of both adaptation and plasticity in three eucalypt species with high gene flow that occupy semi-arid to arid environments in eastern and western Australia [28,29,30]. In contrast, studies at a finer spatial scale (<100 km) have shown mixed results, with some finding very weak signals of adaptation [31,32], while Eckert et al. [33] found strong signals of adaptation to a number of environmental variables in *Pinus lambertia* across just 35 km. Thus, the relationship between scale of gene flow and signals of adaptation appears to be complex and is likely to be influenced by combinations of standing genetic variation, selection strength, genetic connectivity, and spatial scale.

Further analysis in a wider range of species and habitat contexts is needed to provide greater understanding of the extent of climate adaptation and the factors that influence it in plant species. Across the world, maintenance of riparian vegetation is considered critical for healthy functioning of rivers and streams, and their unique ecosystems [34,35]. Restoration of these systems and maintenance of intact vegetation is a focus of management programs [34,36]. Knowledge of adaptation to climate and capacity for plasticity is a key basis for effective design of these management programs [37,38]. Analysis of genetic structure in species in riparian systems has shown that they are not always as genetically connected as has been assumed based on ecological features of rivers [39,40], so prediction of adaptation is likely to be complex and requires further investigation. 

This study investigated signals of selection to climate in two species along the Warren River in south-western Australia, a global biodiversity hotspot [41] that is projected to experience an increase in temperature and a decrease in precipitation, particularly in early winter [42]. *Astartea leptophylla* and *Callistachys lanceolata* have differing distributions and different patterns of genetic structure, gene flow, and connectivity, enabling investigation of the hypothesis that genetic connectivity influences signals of selection over relatively short geographical distance. A previous investigation of neutral genetic structure in *A. leptophylla* showed genetic patterns consistent with high gene flow in the middle and lower catchment, although with greater isolation in the upper catchment due to fragmentation, while populations of *C. lanceolata* had high genetic differentiation consistent with low gene flow throughout the catchment [43]. *Astartea leptophylla* also had lower levels of standing genetic variation (heterozygosity) than *C. lanceolata* [43]. Based on previous theoretical and empirical studies on the interaction between gene flow and selection, we hypothesized that the species with a tight distribution along the river bank will have little signal of selection due to high gene flow and a narrow habitat niche, whereas the species with more widespread distribution that is present on the river bank but also occurs away from the river in wetter habitats, will show a greater signal of adaptation, consistent with higher levels of population differentiation. 

## 2. Materials and Methods 

### 2.1. Study System and Sampling

**Figure 1:**
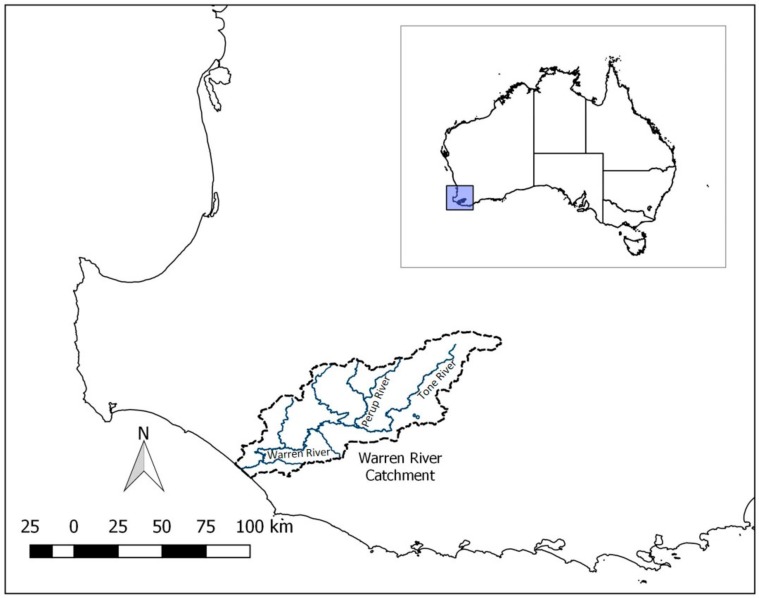
Location of the Warren River and its tributaries in south-western Australia.

The Warren River catchment is in south-western Australia (Figure 1), where the Warren River flows from the confluence of the Tone and Perup Rivers. The river is seasonal, with high flows in the winter months and flow that ceases at the top of the catchment in the dry summer months, when the riverbed in the upper catchment turns into patchy waterholes. 

**Figure 2:**
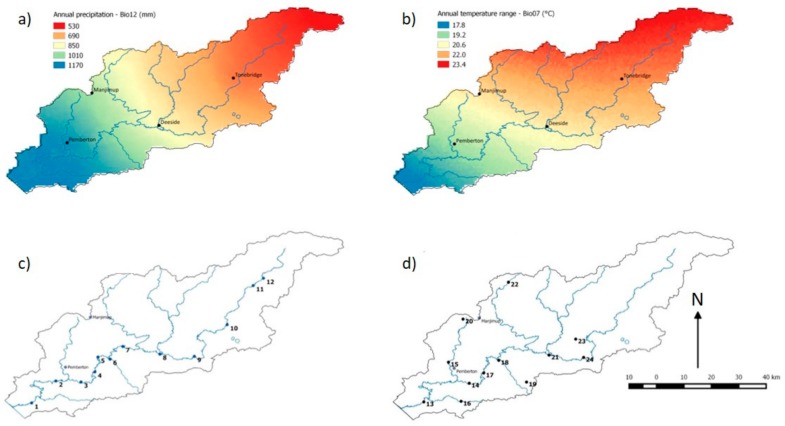
The gradient of (**a**) annual precipitation and (**b**) annual range in temperature across the study catchment with the locations of four weather stations marked. Climate gradients adapted from [44]. Locations of study sites across catchment for (**c**) *Astartea leptophylla* and (**d**) *Callistachys lanceolata*.

**Figure 3:**
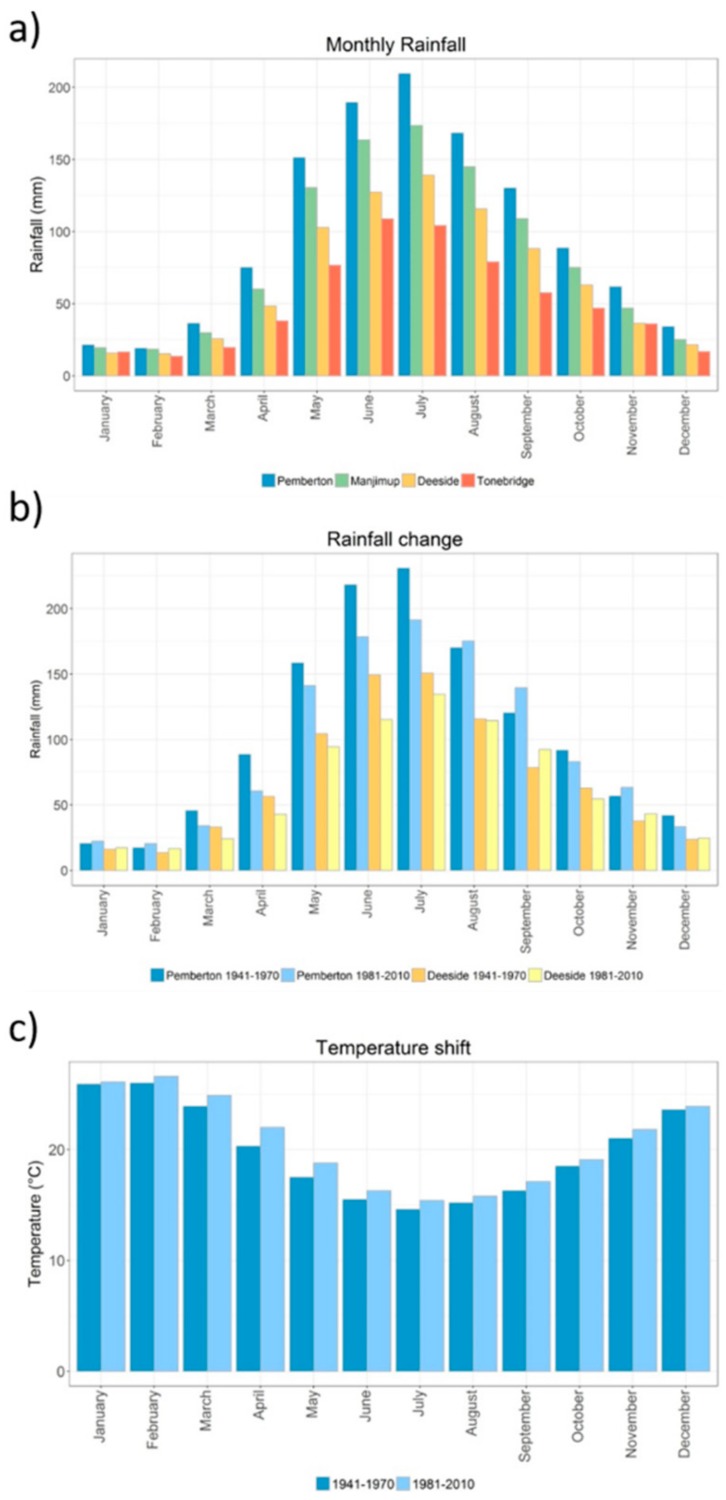
(**a**) Average monthly rainfall at four weather stations across the Warren River catchment (1941–2015), for locations see Figure 2; (**b**) change in rainfall at two weather stations, Pemberton (blue) and Deeside (yellow), from 1941–1970 in darker color and 1981–2010 in lighter color; and (**c**) change in monthly mean maximum temperature at Pemberton from 1941–1970 in darker color and 1981–2010 in lighter color. Adapted from [45].

Across the Warren catchment there is a steep climate gradient from the lower south-west corner to the upper catchment in the north-east. The south-west of the catchment has high rainfall, up to 1200 mm annually, while the drier north-east averages only 510 mm each year (Figure 2a) [44]. The catchment has a strong seasonal rainfall pattern with the majority of precipitation in the middle of the year (May–September) (Figure 3a) [45]. The annual mean temperature does not vary greatly across the catchment (14.4 °C to 15.6 °C). However, there are differences in the annual range of temperature experienced across the catchment (Figure 2b), the north-east has the highest maximum temperatures and the lowest minimums, resulting in a much greater temperature range experienced at the top of the catchment. A change in rainfall and temperature has been documented across the catchment in the last decade. Annual rainfall has decreased, although this is not a consistent pattern across the year with a decrease in May, June, and July (end autumn and early winter), and an increase in November, December, January, and February (late spring and summer) (Figure 3b). The change in temperature has been more consistent throughout the year, with a general increase in temperatures in all months (Figure 3c).

*Astartea leptophylla* and *C. lanceolata* are native to south-western Australia and occur within the Warren River catchment. *Astartea leptophylla* is a shrub that grows to 5 m tall while *C. lanceolata* is a tree or shrub that grows to 8 m tall [46,47]. Both species are insect pollinated with the seed of *A. leptophylla* dispersed via water and wind, and seed of *C. lanceolata* dispersed by gravity. *Astartea leptophylla* is restricted to the banks of rivers that are fast-flowing during the wet winter season [46], and *C. lanceolata* is widespread, generally found in wet areas or along water courses but is not restricted to rivers. *Astartea leptophylla* has high gene flow and low genetic structure consistent with having a continuous distribution along the river bank [43]. *Callistachys lanceolata* has high genetic structure and restricted gene flow, which is also consistent with its more patchy distribution and gravity dispersed seed [43]. *Astartea leptophylla* is also impacted by habitat fragmentation at the north-eastern extent of its range within the catchment. As *A. leptophylla* is restricted to the major water flow systems, sampling for this species occurred along the main Warren River and its tributary the Tone River, where 12 sites were sampled (Figure 2c). *Callistachys lanceolata* does not occur as far upstream on the Warren River as *A. leptophylla*, and the 12 sampled sites were located both on the main river and on tributaries of the Warren River (Figure 2d). At each site, leaf samples were taken from 12 individual plants that were at least 10 meters apart and stored on silica gel in the field before freeze drying. The 12 sites of *A. leptophylla* that were sampled represent an annual rainfall of 562–1130 mm and an annual temperature range of 15.4–16 °C. The 12 sites of *C lanceolata* that were sampled represent an annual rainfall of 724–1136 mm and an annual mean temperature range of 15–16 °C.

### 2.2. Genomic Assay and Environmental Analysis 

Genomic DNA was extracted using a modified CTAB method [48], with the addition of sodium sulfite [49], and 1% *w/v* polyvinylpyrrolidone to the extraction buffer. Following extraction and quantification, DNA samples were sent to Diversity Arrays Technology Pty Ltd, Canberra, for DArTseq analysis. DArTSeq is a genome complexity reduction method that uses a combination of restriction enzymes that separate low copy sequences from the repetitive fraction of the genome [50]. The sequencing results were run through DArT PL’s proprietary single nucleotide polymorphism (SNP)-calling algorithms (DArTsoft14) [51], and the resultant dataset was filtered using the R package dartR 1.1.2 [52] in R [53], to a loci call rate of 95%, individual call rate to 95%, SNPs with a reproducibility score of 1 and Hardy–Weinberg equilibrium with an alpha of 0.05. The R package Poppr 2.8.1 [54] was then used to filter to a minor allele frequency of 2%, and the SNPRelate package 1.16.0 [55] was used to filter for linkage disequilibrium with a ld.threshold of 0.2. Finally, monomorphic loci were removed from the resulting dataset.

Environmental variable values for thirty-one bioclimatic variables for each population were retrieved from the Atlas of Living Australia (Atlas of Living Australia website at http://www.ala.org.au. Accessed 3 November 2015). To reduce redundancy and minimize high correlations, the R package USDM 1.1-18 [56] in R was used to remove the variables that had a correlation above 0.9 in *C. lanceolata*; this removed 22 variables, leaving nine variables for temperature (3), precipitation (3), moisture (2), and radiation (1). The values for variables for each site are listed in the Supplementary Materials Tables S1 and S2 and FS9. As we wanted to compare results between species, the same variables were also used for *A. leptophylla*, although all variables for *A. leptophylla* were highly correlated. 

To elucidate genotype environment associations, the Bayesian hierarchical model implemented in BayPass was used as it corrects for demographic effects and has been shown to be among the most efficient at identifying true positives [57]. Initially, the core model in BayPass was run four times with default settings, with a nval of 100,000, burnin of 50,000, npilot of 30, and pilotlength of 5000, results were averaged over runs. Calibration of the XtX statistic was undertaken following the manual by using the function simulate.baypass to create a pseudo-observed dataset, and subsequently run using the same settings on the core model to calculate a 3% and 97% threshold to discriminate between neutral and outlier loci. SNPs having XtX statistics above the 97% and below the 3% threshold, representing the directional and balancing selection respectively, were considered outliers. 

BayPass was used to identify associations between these outlier loci and the environmental gradients. The core model was run with the neutral dataset (both directional and balancing removed from dataset), each run was 100,000, with a burnin of 50,000 and 30 pilot runs of 5000, this was repeated four times, and the mean of the four runs was taken to create the neutral covariance matrix. The auxiliary model was run using the neutral covariance matrix and the nine environmental variables. Each run was 100,000, with a burnin of 50,000 and 30 pilot runs of 5000, and this was repeated four times and the mean of the four runs was taken as the final results. Correlations with a Bayes factor of more than 20 were considered to have strong evidence for associations (as proposed in Kass and Raftery 1995) [58], as the Bayes factor is the support for the alternative model (the corrected allele frequency correlation with the environmental variable) compared to the null model (the neutral covariance matrix). 

The data obtained in this study used newer sequencing methods that have been developed and refined since the earlier analysis of genetic structure in these species [43] and was used to confirm the results of the previous analysis. Analysis of genetic structure and differentiation were carried out using the neutral dataset, created after removing the identified directional and balancing outlier loci, to confirm the earlier findings and verify the patterns of diversity to provide the context for evaluation in this study. To identify clusters of individuals and visualize the major axes of variation between clusters, principal coordinates analysis was undertaken, as well as estimation of genetic diversity characteristics, private allele number, expected and observed heterozygosity, and inbreeding coefficients, implemented in the adegenet 2.1.1 [59], Poppr 2.8.1 [54], and Hierfstat 0.04-22 [60] packages in R. Population genetic structure of each species was explored using Structure 2.3.4 [61] using the neutral data set obtained after filtering and outlier removal. Analysis using K-values from 1 to 14 were undertaken for each dataset. Ten independent runs were undertaken for each K-value with a burnin of 50,000 and 250,000 Markov Chain Monte Carlo (MCMC) iterations. The R package pophelper 2.2.9 [62] was used to visualize results and select the most probable K based on the ∆K metric. Pairwise F_ST_ value were estimated using the R package hierfstat 0.04-22 [60].

## 3. Results

The results from the DArT pipeline analysis resulted in 93,076 and 16,542 SNPs for *A. leptophylla* and *C. lanceolate*, respectively. Further filtering to remove loci and samples with high proportions of missing data including loci with minor allele frequencies below 2%, resulted in a final SNP dataset for further analysis consisting of 11,769 SNPs for *A. leptophylla* and 5,331 SNPs for *C. lanceolata*. Outlier analysis in BayPass identified 357 directional and 366 balancing SNPs for *A. leptophylla* and 134 directional and 111 balancing SNPs for *C. lanceolata*. Removing these outlier SNPs from the full dataset resulted in neutral datasets with 10,581 and 4887 SNPs for *A. leptophylla* and *C. lanceolate*, respectively.

### 3.1. Genetic Differentiation and Population Structure

While the new sequencing methods showed very similar results to the previous study [43], there were some minor differences. The genetic differentiation results confirmed lower differentiation in *A. leptophylla* that had pairwise population F_ST_ values from 0.01 to 0.078, compared to values for *C. lanceolata* that ranged from 0.039 to 0.417. Populations of *A. leptophylla* in the upper catchment (A10, A11, A12) showed higher differentiation, both among populations in the upper catchment and between these populations and those in the middle and lower catchment (Supplementary Materials Figure S1). Population diversity characteristics were consistent with the previous work, showing lower heterozygosity and fewer private alleles in *A. leptophylla* than in *C. lanceolata*. There were no private alleles seen in populations A1–A9 of *A. leptophylla* in the lower and middle catchment, highlighting the extent of gene flow occurring amongst these populations (Supplementary Materials Figures S4 and S8).

Principal component analysis supported these patterns, as populations A10, A11, and A12 of *A. leptophylla* were highly differentiated from the other sites in the middle and lower catchment; however only a small amount of variation is explained by the axes. When these populations were removed to focus on the genetic structure present in the lower and middle catchment, populations A1, A2, and A3 grouped together and populations A8 and A9 grouped together, and populations A4, A5, A6, and A7 forming a loose cluster (Supplementary Materials Figures S2 and S3). Principal component analysis for *C. lanceolata* showed high differentiation of the four most inland populations (C21–24) from the remaining populations (Figure S6). Finer scale analysis after removal of these four populations showed limited differentiation among the remaining populations in the lower catchment (Figure S7).

**Figure 4:**
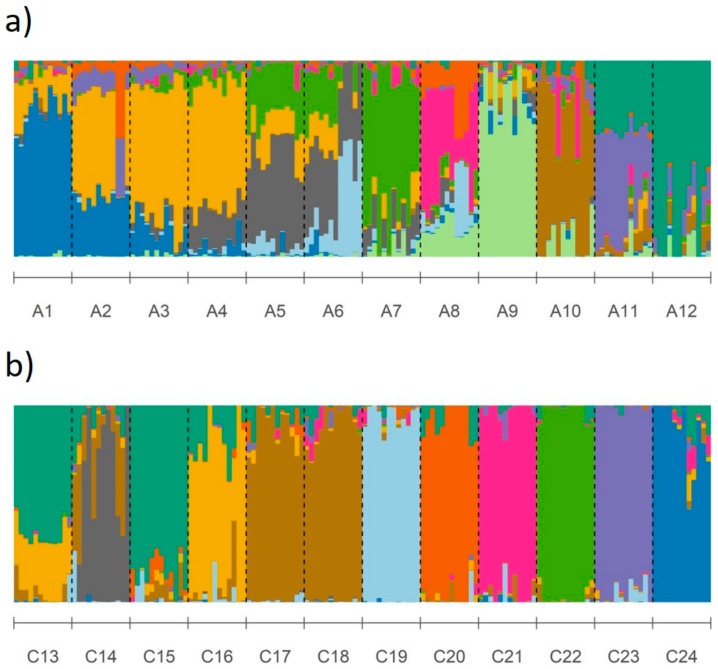
STRUCTURE plots for identified optimal number of genetic clusters for (**a**) *Astartea leptophylla* (K = 11) and (**b**) *Callistachys lanceolata* (K = 10), with each color representing a genetic cluster.

The STRUCTURE results for *A. leptophylla* showed higher number of clusters (K = 11) than in the previous study but still showed high admixture among populations, particularly in populations in the lower and middle catchment (Figure 4a). The STRUCTURE results for *C. lanceolata* were similar to previous results and identified ten genetic clusters that were generally representative of populations, with low levels of admixture, particularly in the middle and upper catchment (Figure 4b). 

### 3.2. Environmental Associations

**Figure 5:**
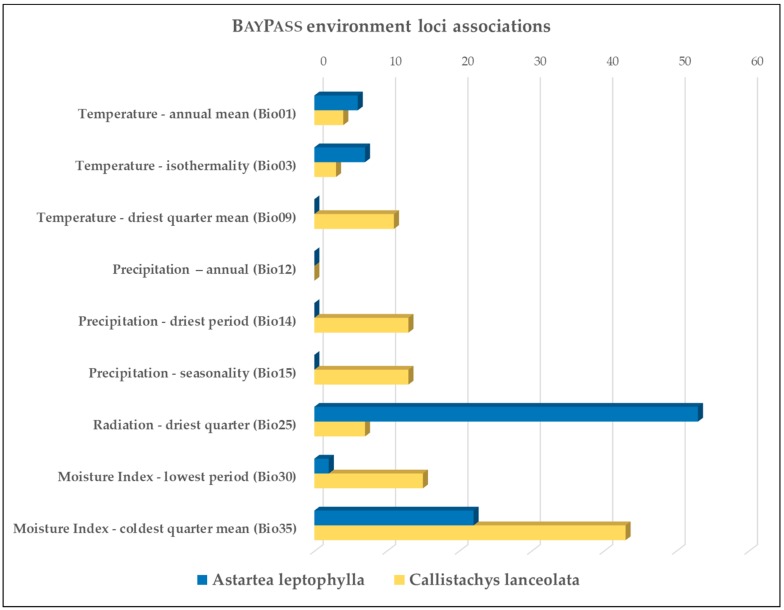
The number of environmental loci associations detected in BayPass for *Astartea leptophylla* and *Callistachys lanceolata* for each environmental variable.

**Figure 6:**
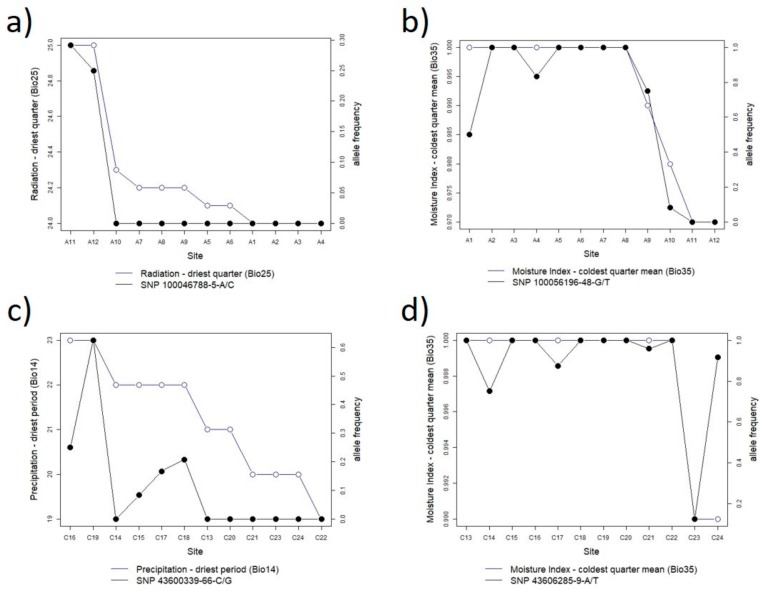
The strongest correlations for *Astartea leptophylla* with (**a**) radiation of the driest quarter and (**b**) moisture index of the coldest quarter; and the two strongest correlations for *Callistachys lanceolata* with (**c**) precipitation of the driest period and (**d**) moisture index of the coldest quarter mean.

The BayPass analysis of *A. leptophylla* identified 88 loci with significant correlations with an environmental variable. Only two of these loci were associated with multiple variables, each having two significant correlations. The majority of associations (83%) were with two environmental variables, radiation of the driest quarter, and moisture index of the coldest quarter, and only small numbers of correlations with other environmental variables (Figure 5). A closer look at these variables and their strongest correlations show a strong divide between those sites in the lower and middle catchment and those in the upper catchment (Figure 6a,b). This is particularly the case with radiation of the driest quarter where the strongest correlation had fixation for allele A in sites A1–A10, while allele C was only present in sites A11 and A12 (Figure 6a). The top ten correlations (above 0.9, with the highest at 0.99) consisted of four with moisture index of the coldest quarter mean, five with radiation of the driest quarter, and one with temperature isothermality.

The BAYPASS analysis of *C. lanceolata* identified 109 loci with significant correlations with an environmental variable, none of the loci were associated with more than one variable. Moisture index of the coldest quarter had the highest number of associations, while four other environmental variables had moderate numbers of correlations being moisture index of the lowest period (15), precipitation of the driest period (13), seasonality of precipitation (13), and mean temperature of the driest quarter (11), all variables related to water availability (Figure 5). Moisture index of the coldest quarter was also included in six of the top 10 correlations, the highest correlation being 0.95 (Figure 6c). The other top 10 correlations were with precipitation of the driest quarter (1), with temperature of the driest quarter (2), and with seasonality of precipitation (1), and only the top three correlations were above 0.9, while the rest ranged from 0.81–0.86.

The highest number of associations for *C. lanceolata* was with the variable mean moisture index of the coldest quarter, and while this variable produced the most and often strongest correlations, these results should be taken with caution as there was only two different values for the variable in this species and as such many of the sites represent pseudo replication and the correlations are between only two points (Figure 6d).

## 4. Discussion

Investigation of signals of selection in two riparian species that occur along the same river system suggest a strong influence of gene flow and standing genetic variation on signals of selection. A smaller number of correlations with climate variables in the restricted riverbank species that has high gene flow and lower heterozygosity is consistent with the hypothesis of genetic connectivity mixing genomes and minimizing development of adaptation to climate as well as the need for high levels of standing genetic variation for the development of adaptation. The impact of fragmentation in isolating some populations has likely accelerated the development of signals of selection between loci and those environmental variables that have a high selective pressure on those isolated populations. In contrast, the widespread but more patchily distributed species that has lower gene flow and higher heterozygosity showed the predicted correlations with climate variables due to reduced genetic connectivity and high level of standing genetic variation leading to development of signals of selection in populations. 

### 4.1. Signals of Selection

The results of the environmental association analysis in *A. leptophylla* showed a separation or break between populations in the lower and middle catchment with those in the upper catchment. This is also reflected in the neutral population structure, which has been attributed to fragmentation that has most likely isolated the populations in the upper catchment and reduced the gene flow between these sites and those lower in the catchment. These overlying patterns suggest that environmental variables with strong selection pressures combined with recent inhibition of gene flow may lead to a signal of adaptation in this more stressful environment compared to little signal of adaptation on the more connected populations of the lower and middle catchment. 

In contrast, genetic analysis in the more widespread *C. lanceolata* found more correlations with environmental variables, as expected from a species with a patchier distribution and concomitant patterns of genetic differentiation. The strongest correlations and the majority of associations involving a moisture or precipitation variable, suggesting that precipitation is an important factor for this species, and that the timing of precipitation may be significant in enabling persistence through the summer months when rainfall is very low. 

### 4.2. Selection, Distribution and Gene Flow

The lack of strong signals of selection to most variables across the climatic gradients in *A. leptophylla* in the Warren River catchment might reflect its historical habitat and distribution. *Astartea leptophylla* is restricted to the banks of the main rivers where there is less reliance on rainfall for moisture availability, and the microclimate created in this riparian habitat may reduce the effect of temperature variables that are modeled over larger scales. This decoupling of local and regional climatic features, that is facilitated through temperature modulation and lack of reliance on rainfall by vegetation restricted to the riverbank, would likely be consistent along the length of the river thus negating opportunities for development of local adaptation in the face of sustained gene flow.

Analysis of neutral genetic structure in *A. leptophylla* showed genetic patterns that are consistent with high levels of gene flow in the lower and middle catchment and low standing genetic variation. Low genetic structure and high connectivity amongst populations would reduce adaptation to the local environment through continual immigration of alleles [63] from both upper and lower sites, representing warmer and cooler environments. High gene flow within a species also allows the standing genetic diversity within populations to be maintained or increased [63]. The limited development of local adaptation within *A. leptophylla*, is likely due to the combination of occurrence in a microclimate created along riverbanks, high gene flow in the middle and lower catchment, and low standing genetic variation. Thus, there are multiple interacting facets of environment, gene flow and genetic variation that contribute to the lack of a signal of selection in a riparian species restricted to riverbanks where connectivity has been maintained through intact vegetation communities across much of its range. 

In contrast to *A. leptophylla*, the more widely distributed *C. lanceolata* showed a strong signal of selection to a number of bioclimatic variables, particularly precipitation and moisture variables. Analysis of neutral genetic diversity confirmed *C. lanceolata* has high levels of genetic differentiation consistent with low levels of gene flow throughout the catchment and high levels of standing genetic variation, factors that provide conditions for development of selection across a climatic gradient [8]. The identification of signals of selection associated with bioclimatic variables across the environmental gradient of the Warren River catchment in *C. lanceolata* is consistent with its genetic structure and low levels of gene flow. These results demonstrate that the interacting facets of environment, gene flow, and genetic structure influence development of selection, in this case contributing to a signal of selection in this riparian species that also has a wider but patchy distribution in wetter areas.

High gene flow is considered to reduce opportunities for selection and therefore limit development of local adaptation [21,64], yet evaluation of the role of gene flow in development of adaptation requires a more detailed understanding of the spatial scale of heterogeneity and the types of selection pressure involved [64]. Despite the predicted influence of gene flow in reducing development of adaptation, a number of studies across large distances (1000–2500 km) have found signals of local adaptation to climate variables in species with continuous distributions and assumed high gene flow due to wind pollination (e.g. Conifer [22], Spruce [23,65], Pine [25,26]). This suggests further investigation is required into the spatial scale of local adaptation in species with large ranges across heterogeneous landscapes [33]. This complexity is also evident over much shorter distances (35–65 km), with both weak and strong signals of local adaptation being found in pines that may be assumed to have high gene flow as they are wind pollinated [31,33]. It appears likely that over short distances, high gene flow can mitigate the selection pressures that might cause local adaptation, but at greater distance with stronger heterogeneity across the range, and where gene flow may be high but not panmictic, selection pressures are great enough to allow development of local adaptation. The current study investigated signals of selection over relatively short distance of 100 km and found differing responses in species with differing population structure and gene flow. The species with high gene flow, *A. leptophylla*, showed very weak signals of selection for climate variables in populations in the majority of the distribution, although more restricted gene flow associated with fragmentation led to evidence for some adaptation. In contrast, the species with low gene flow, *C. lanceolata*, showed the expected signals of selection even over a relatively short distance. Thus, our results are consistent with expectations from the hypothesis that levels of adaptation are influenced by a combination of the level and scale of gene flow and the strength of the selection pressure [21]. 

### 4.3. Implications for Persistence

Climate change is already having an impact on species in the Warren catchment, as a recent study has shown reduced recruitment in species at the extreme boundary of the upper catchment [66] indicating a contraction at the warm, dry end of the range. While it is not likely to be feasible to prevent this contraction, the opportunity for selection of genotypes with greater adaptation to warmer climates can be increased through collection and storage of seed for these populations and subsequent use of this seed in climate adaptation strategies, such as assisted gene migration and climate adjusted provenancing that move seed into populations in the direction of projected climate change [10,11]. 

The lack of apparent selection to climatic factors among populations of *A. leptophylla* across its range in the Warren River catchment might suggest that predicted changes in climate may not adversely affect species persistence, provided standing genetic variation is maintained through high levels of genetic connectivity across the catchment. However, fragmentation in the extreme warm, dry end of the catchment has reduced gene flow between populations allowing development of signals of adaptation in those environmental variables with strong selection pressure. This was seen in some loci with change in fixation of alleles over short distance in populations in the upper catchment. While there is high gene flow in the lower catchment for this species and the standing genetic variation may be adequate to cope with expected changes in climate, assisted gene migration/climate-adjusted provenancing [10,11] may also be required to ensure continuity of historic high levels of gene flow and possibly help to increase standing genetic variation in the face of habitat fragmentation. 

*Callistachys lanceolata* is more restricted in its distribution across the catchment than *A. leptophylla* and occurs in the cooler wetter two thirds of the catchment. The signals of selection that were detected in this species were to climate variables associated with moisture availability during extreme time periods (moisture of the lowest period, precipitation of the driest period). This may indicate that climatic conditions at the extreme dry end of the range are a limiting factor in the distribution of the species, and therefore predicted climatic shifts will reduce suitable habitat for this species. While *C. lanceolata* occurs across a narrower annual rainfall range than *A. leptophylla*, it is not as restricted in its habitat niche, occurring in wet areas but not restricted to riverbanks. Greater isolation and less connectivity among populations is likely to limit the natural flow of genes adapted to the predicted drier and hotter climates. Thus, assisted gene migration/climate-adjusted provenancing [10,11] would be a climate adaptation strategy that would enhance gene flow and maintain the genetic diversity required for adaptation to changing climates.

## 5. Conclusions

Differing gene flow, standing genetic variation, and habitat context in the two species studied here suggest patterns of signals of selection consistent with theoretical predictions of the effects of genetic connectivity on adaptation. The combination of riparian microclimate, higher levels of connectivity, and low standing genetic variation mitigated the development of selection in most environmental variables for *A. leptophylla*, while those environmental variables with a strong selection pressure have formed signals of selection due to the isolation and reduced geneflow of sites in the upper catchment. In contrast, the less specific habitat requirement of *C. lanceolata* combined with low gene flow and higher standing genetic variation was consistent with conditions allowing development of selection to bioclimatic variables. While it is difficult to control environmental factors, our study on two species with differing levels of connectivity across the same climate gradient has provided the opportunity to test the interaction between genetic connectivity and signals of selection. These findings extend our current understanding on the factors that influence selection across climate gradients, confirming that gene flow influences with signatures of selection. These results also verify the need for management strategies that support genetic connectivity and, when needed, intervention to assist migration of genes to maximize the resilience of populations and species persistence in the face of changing climates.

## Supplementary Materials

The following are available online at https://www.mdpi.com/2073-4425/10/8/579/s1, Table S1: The thirty-one bioclimatic variables and the values for each of the *Astartea leptophylla* sites, Table S2: The thirty-one bioclimatic variables and the values for each of the *Callistachys lanceolata* sites, Figure S1: Pairwise (a) genetic distance (F_ST_) and (b) geographic distance (km) among 12 sites for *Astartea leptophylla*., Figure S2: Principal component analysis of *Astartea leptophylla* populations to identify genetic structure present including (a) axis 1 and 2, and (b) axis 1 and 3. Populations A10, A11, and A12 are highly differentiation from the other sites in the middle and lower catchment; however only a small amount of variation is explained by the axes, Figure S3: Principal component analysis of subset of *Astartea leptophylla* populations, the highly differentiated populations A10, A11, and A12 have been removed to focus on the genetic structure present in the lower and middle catchment, including (a) axis 1 and 2, and (b) axis 1 and 3. The finer look at these populations identifies populations A1, A2, and A3 grouping together in the negative space of the first axis and populations A8 and A9 grouping in the negative space of the second axis, Figure S4: Genetic diversity characteristics for *Astartea leptophylla* populations including, (a) number of private alleles, (b) expected heterozygosity, (c) observed heterozygosity, and (d) inbreeding coefficient, Figure S5: Pairwise (a) genetic distance (F_ST_) and (b) geographic distance (km) among 12 sites for *Callistachys lanceolata*, Figure S6: Principal component analysis of *Callistachys lanceolata* populations to identify genetic structure present including (a) axis 1 and 2, and (b) axis 1 and 3. Populations C23 and C24 are highly differentiation in the first and second axes, while populations C21 and C22 are differentiated from the other sites along the third axis, Figure S7: Principal component analysis of subset of *Callistachys lanceolata* populations, the highly differentiated populations C21. C22. C23 and C24 have been removed to focus on the genetic structure present in the remaining populations, including (a) axis 1 and 2, and (b) axis 1 and 3. The finer look at these populations identifies population C14 differentiated on the first axis and populations C19 and C20 grouping together in the positive space of the second axis, Figure S8: Genetic diversity characteristics for Callistachys lanceolata populations including, (a) number of private alleles, (b) expected heterozygosity, (c) observed heterozygosity, and (d) inbreeding coefficient.
