# Supplementary material for: Gene Flow and Genetic Variation Explain Signatures of Selection across a Climate Gradient in Two Riparian Species
Source: Genes (Basel). 2019 Jul 31;10(8):579. doi: 10.3390/genes10080579 (PMC6723506; doi:10.3390/genes10080579)

## Supplementary Materials

**Table S1.** The thirty-one bioclimatic variables and the values for each of the *Astartea leptophylla* sites.

| Astartea | 1 | 2 | 3 | 4 | 5 | 6 | 7 | 8 | 9 | 10 | 11 | 12 |
|---|---|---|---|---|---|---|---|---|---|---|---|---|
| Temperature - annual mean (Bio01) | 16.000 | 15.800 | 15.700 | 15.800 | 15.700 | 15.700 | 15.700 | 15.600 | 15.500 | 15.400 | 15.400 | 15.400 |
| Temperature - diurnal range mean (Bio02) | 9.700 | 10.200 | 10.400 | 10.700 | 10.900 | 11.000 | 11.300 | 11.400 | 11.400 | 11.900 | 12.300 | 12.300 |
| Temperature - isothermality (Bio03) | 0.550 | 0.540 | 0.540 | 0.540 | 0.530 | 0.530 | 0.530 | 0.530 | 0.530 | 0.520 | 0.520 | 0.520 |
| Temperature - seasonality (Bio04) | 0.970 | 1.050 | 1.080 | 1.110 | 1.160 | 1.160 | 1.200 | 1.220 | 1.240 | 1.310 | 1.380 | 1.390 |
| Temperature - warmest period max (Bio05) | 25.600 | 26.400 | 26.700 | 27.100 | 27.500 | 27.500 | 27.900 | 28.000 | 28.100 | 28.800 | 29.300 | 29.400 |
| Temperature - coldest period min (Bio06) | 8.200 | 7.600 | 7.400 | 7.200 | 7.000 | 7.000 | 6.700 | 6.500 | 6.400 | 6.000 | 5.600 | 5.500 |
| Temperature - annual range (Bio07) | 17.500 | 18.800 | 19.300 | 19.900 | 20.500 | 20.600 | 21.300 | 21.500 | 21.700 | 22.800 | 23.700 | 23.800 |
| Temperature - wettest quarter mean (Bio08) | 13.100 | 12.400 | 12.300 | 12.200 | 12.000 | 12.000 | 11.800 | 11.600 | 11.500 | 11.200 | 10.900 | 10.900 |
| Temperature - driest quarter mean (Bio09) | 19.600 | 19.800 | 19.800 | 20.000 | 20.100 | 20.100 | 20.300 | 20.200 | 20.200 | 20.400 | 20.600 | 20.600 |
| Temperature - warmest quarter (Bio10) | 19.700 | 19.800 | 19.800 | 20.000 | 20.100 | 20.100 | 20.300 | 20.200 | 20.200 | 20.400 | 20.600 | 20.700 |
| Temperature - coldest quarter mean (Bio11) | 12.600 | 12.200 | 12.000 | 11.900 | 11.700 | 11.700 | 11.500 | 11.400 | 11.200 | 10.900 | 10.700 | 10.700 |
| Precipitation - annual (Bio12) | 1130.000 | 1109.000 | 1072.000 | 999.000 | 933.000 | 911.000 | 831.000 | 791.000 | 748.000 | 640.000 | 574.000 | 562.000 |
| Precipitation - wettest period (Bio13) | 48.000 | 46.000 | 45.000 | 42.000 | 39.000 | 38.000 | 35.000 | 33.000 | 31.000 | 25.000 | 22.000 | 22.000 |
| Precipitation - seasonality (Bio15) | 71.000 | 69.000 | 68.000 | 67.000 | 67.000 | 66.000 | 65.000 | 64.000 | 62.000 | 60.000 | 58.000 | 58.000 |
| Precipitation - wettest quarter (Bio16) | 560.000 | 537.000 | 513.000 | 475.000 | 441.000 | 430.000 | 389.000 | 366.000 | 341.000 | 288.000 | 256.000 | 250.000 |
| Precipitation - driest quarter (Bio17) | 64.000 | 66.000 | 66.000 | 62.000 | 60.000 | 59.000 | 56.000 | 56.000 | 56.000 | 54.000 | 53.000 | 52.000 |
| Precipitation - warmest quarter (Bio18) | 66.000 | 68.000 | 67.000 | 64.000 | 61.000 | 60.000 | 57.000 | 56.000 | 56.000 | 54.000 | 53.000 | 52.000 |
| Precipitation - coldest quarter (Bio19) | 534.000 | 518.000 | 499.000 | 463.000 | 431.000 | 419.000 | 380.000 | 358.000 | 335.000 | 281.000 | 249.000 | 243.000 |
| Radiation - annual mean (Bio20) | 16.300 | 16.300 | 16.300 | 16.400 | 16.600 | 16.600 | 16.800 | 16.800 | 16.800 | 17.100 | 17.300 | 17.400 |
| Radiation - highest period (Bio21) | 26.900 | 26.800 | 26.800 | 26.900 | 27.000 | 27.000 | 27.100 | 27.100 | 27.100 | 27.200 | 27.300 | 27.300 |
| Radiation - lowest period (Bio22) | 7.200 | 7.300 | 7.300 | 7.400 | 7.500 | 7.500 | 7.600 | 7.600 | 7.700 | 7.900 | 8.000 | 8.100 |
| Radiation - seasonality (Bio23) | 42.000 | 42.000 | 42.000 | 41.000 | 41.000 | 41.000 | 40.000 | 40.000 | 40.000 | 39.000 | 39.000 | 38.000 |
| Radiation - wettest quarter (Bio24) | 8.100 | 8.300 | 8.400 | 8.500 | 8.600 | 8.600 | 8.700 | 8.800 | 8.800 | 9.100 | 9.300 | 9.300 |
| Radiation - driest quarter (Bio25) | 24.000 | 24.000 | 24.000 | 24.000 | 24.100 | 24.100 | 24.200 | 24.200 | 24.200 | 24.300 | 25.000 | 25.000 |
| Radiation - warmest quarter (Bio26) | 23.200 | 23.200 | 23.200 | 23.300 | 23.400 | 23.400 | 23.500 | 24.200 | 24.200 | 24.300 | 24.400 | 24.400 |
| Radiation - coldest quarter (Bio27) | 9.200 | 9.300 | 9.300 | 9.400 | 9.500 | 9.600 | 9.700 | 9.800 | 9.800 | 10.100 | 10.300 | 10.400 |
| Moisture Index - annual mean (Bio28) | 0.680 | 0.680 | 0.680 | 0.660 | 0.650 | 0.650 | 0.640 | 0.630 | 0.620 | 0.590 | 0.570 | 0.560 |
| Moisture Index - lowest period (Bio30) | 0.090 | 0.090 | 0.090 | 0.090 | 0.080 | 0.080 | 0.080 | 0.080 | 0.070 | 0.070 | 0.070 | 0.060 |
| Moisture Index - seasonality (Bio31) | 56.000 | 56.000 | 56.000 | 58.000 | 59.000 | 60.000 | 62.000 | 62.000 | 63.000 | 66.000 | 69.000 | 69.000 |
| Moisture Index - warmest quarter mean (Bio34) | 0.120 | 0.130 | 0.130 | 0.120 | 0.110 | 0.110 | 0.100 | 0.100 | 0.100 | 0.090 | 0.090 | 0.090 |
| Moisture Index - coldest quarter mean (Bio35) | 1.000 | 1.000 | 1.000 | 1.000 | 1.000 | 1.000 | 1.000 | 1.000 | 0.990 | 0.980 | 0.970 | 0.970 |

## Supplementary Materials

**Table S2**. The thirty-one bioclimatic variables and the values for each of the *Callistachys lanceolata* sites.

| Callistachys | 13 | 14 | 15 | 16 | 17 | 18 | 19 | 20 | 21 | 22 | 23 | 24 |
|---|---|---|---|---|---|---|---|---|---|---|---|---|
| Temperature - annual mean (Bio01) | 16.000 | 15.700 | 15.700 | 15.700 | 15.800 | 15.700 | 15.200 | 15.000 | 15.600 | 15.000 | 15.100 | 15.500 |
| Temperature - diurnal range mean (Bio02) | 9.700 | 10.400 | 10.500 | 10.000 | 10.700 | 11.000 | 10.700 | 11.100 | 11.400 | 12.200 | 11.500 | 11.400 |
| Temperature - isothermality (Bio03) | 0.550 | 0.540 | 0.540 | 0.550 | 0.540 | 0.530 | 0.530 | 0.520 | 0.530 | 0.520 | 0.520 | 0.530 |
| Temperature - seasonality (Bio04) | 0.970 | 1.080 | 1.100 | 1.020 | 1.110 | 1.160 | 1.160 | 1.220 | 1.220 | 1.350 | 1.280 | 1.240 |
| Temperature - warmest period max (Bio05) | 25.700 | 26.700 | 26.900 | 26.000 | 27.100 | 27.500 | 27.000 | 27.500 | 28.000 | 28.700 | 28.100 | 28.100 |
| Temperature - coldest period min (Bio06) | 8.100 | 7.400 | 7.200 | 7.700 | 7.200 | 7.000 | 6.700 | 6.200 | 6.500 | 5.300 | 6.000 | 6.400 |
| Temperature - annual range (Bio07) | 17.600 | 19.300 | 19.600 | 18.300 | 19.900 | 20.600 | 20.300 | 21.200 | 21.500 | 23.400 | 22.100 | 21.700 |
| Temperature - wettest quarter mean (Bio08) | 13.100 | 12.300 | 12.100 | 12.500 | 12.200 | 12.000 | 11.500 | 11.100 | 11.600 | 10.600 | 11.000 | 11.500 |
| Temperature - driest quarter mean (Bio09) | 19.700 | 19.800 | 19.800 | 19.600 | 20.000 | 20.100 | 19.600 | 19.600 | 20.200 | 20.100 | 19.900 | 20.200 |
| Temperature - warmest quarter (Bio10) | 19.700 | 19.800 | 19.900 | 19.600 | 20.000 | 20.100 | 19.600 | 19.700 | 20.200 | 20.100 | 20.000 | 20.200 |
| Temperature - coldest quarter mean (Bio11) | 12.600 | 12.000 | 11.900 | 12.200 | 11.900 | 11.700 | 11.200 | 10.800 | 11.400 | 10.400 | 10.700 | 11.200 |
| Precipitation - annual (Bio12) | 1126.000 | 1072.000 | 1078.000 | 1136.000 | 999.000 | 911.000 | 956.000 | 957.000 | 791.000 | 744.000 | 724.000 | 748.000 |
| Precipitation - wettest period (Bio13) | 48.000 | 45.000 | 45.000 | 48.000 | 42.000 | 38.000 | 40.000 | 41.000 | 33.000 | 32.000 | 29.000 | 31.000 |
| Precipitation - seasonality (Bio15) | 71.000 | 68.000 | 68.000 | 69.000 | 67.000 | 66.000 | 65.000 | 68.000 | 64.000 | 67.000 | 62.000 | 62.000 |
| Precipitation - wettest quarter (Bio16) | 557.000 | 513.000 | 518.000 | 551.000 | 475.000 | 430.000 | 445.000 | 459.000 | 366.000 | 357.000 | 330.000 | 341.000 |
| Precipitation - driest quarter (Bio17) | 64.000 | 66.000 | 65.000 | 67.000 | 62.000 | 59.000 | 64.000 | 60.000 | 56.000 | 52.000 | 56.000 | 56.000 |
| Precipitation - warmest quarter (Bio18) | 66.000 | 67.000 | 67.000 | 69.000 | 64.000 | 60.000 | 66.000 | 62.000 | 56.000 | 53.000 | 56.000 | 56.000 |
| Precipitation - coldest quarter (Bio19) | 531.000 | 499.000 | 502.000 | 533.000 | 463.000 | 419.000 | 438.000 | 450.000 | 358.000 | 346.000 | 323.000 | 335.000 |
| Radiation - annual mean (Bio20) | 16.300 | 16.300 | 16.400 | 16.200 | 16.400 | 16.600 | 16.500 | 16.700 | 16.800 | 17.100 | 16.900 | 16.800 |
| Radiation - highest period (Bio21) | 26.900 | 26.800 | 26.800 | 26.800 | 26.900 | 27.000 | 26.900 | 27.000 | 27.100 | 27.300 | 27.100 | 27.100 |
| Radiation - lowest period (Bio22) | 7.200 | 7.300 | 7.400 | 7.200 | 7.400 | 7.500 | 7.400 | 7.600 | 7.600 | 7.800 | 7.700 | 7.700 |
| Radiation - seasonality (Bio23) | 42.000 | 42.000 | 42.000 | 42.000 | 41.000 | 41.000 | 41.000 | 41.000 | 40.000 | 40.000 | 40.000 | 40.000 |
| Radiation - wettest quarter (Bio24) | 8.100 | 8.400 | 8.400 | 8.300 | 8.500 | 8.600 | 8.500 | 8.600 | 8.800 | 9.000 | 8.900 | 8.800 |
| Radiation - driest quarter (Bio25) | 24.000 | 24.000 | 24.000 | 23.900 | 24.000 | 24.100 | 24.000 | 24.800 | 24.200 | 25.100 | 24.900 | 24.200 |
| Radiation - warmest quarter (Bio26) | 23.200 | 23.200 | 23.300 | 23.200 | 23.300 | 23.400 | 23.300 | 23.500 | 24.200 | 24.400 | 24.200 | 24.200 |
| Radiation - coldest quarter (Bio27) | 9.200 | 9.300 | 9.400 | 9.200 | 9.400 | 9.600 | 9.400 | 9.600 | 9.800 | 10.000 | 9.900 | 9.800 |
| Moisture Index - annual mean (Bio28) | 0.680 | 0.680 | 0.680 | 0.680 | 0.660 | 0.650 | 0.670 | 0.660 | 0.630 | 0.620 | 0.620 | 0.620 |
| Moisture Index - lowest period (Bio30) | 0.090 | 0.090 | 0.100 | 0.090 | 0.090 | 0.080 | 0.090 | 0.090 | 0.080 | 0.070 | 0.080 | 0.070 |
| Moisture Index - seasonality (Bio31) | 56.000 | 56.000 | 56.000 | 55.000 | 58.000 | 60.000 | 57.000 | 58.000 | 62.000 | 64.000 | 63.000 | 63.000 |
| Moisture Index - warmest quarter mean (Bio34) | 0.120 | 0.130 | 0.130 | 0.130 | 0.120 | 0.110 | 0.120 | 0.120 | 0.100 | 0.090 | 0.100 | 0.100 |
| Moisture Index - coldest quarter mean (Bio35) | 1.000 | 1.000 | 1.000 | 1.000 | 1.000 | 1.000 | 1.000 | 1.000 | 1.000 | 1.000 | 0.990 | 0.990 |

## Supplementary Materials

**Figure S1**. Pairwise a) genetic distance ($F_{ST}$) and b) geographic distance (km) among 12 sites for *Astartea leptophylla*.

a)

**Pairwise genetic distance (FST)**

| Site | A1 | A2 | A3 | A4 | A5 | A6 | A7 | A8 | A9 | A10 | A11 | A12 |
|---|---|---|---|---|---|---|---|---|---|---|---|---|
| A12 | | | | | | | | | | | | 0 |
| A11 | | | | | | | | | | | 0 | 0.07 |
| A10 | | | | | | | | | | 0 | 0.07 | 0.07 |
| A9 | | | | | | | | | 0 | 0.05 | 0.06 | 0.07 |
| A8 | | | | | | | | 0 | 0.03 | 0.05 | 0.07 | 0.07 |
| A7 | | | | | | | 0 | 0.03 | 0.03 | 0.05 | 0.06 | 0.07 |
| A6 | | | | | | 0 | 0.02 | 0.03 | 0.04 | 0.06 | 0.07 | 0.07 |
| A5 | | | | | 0 | 0.01 | 0.02 | 0.03 | 0.03 | 0.05 | 0.06 | 0.07 |
| A4 | | | | 0 | 0.01 | 0.02 | 0.02 | 0.03 | 0.03 | 0.05 | 0.06 | 0.07 |
| A3 | | | 0 | 0.01 | 0.02 | 0.02 | 0.02 | 0.03 | 0.03 | 0.05 | 0.06 | 0.07 |
| A2 | | 0 | 0.01 | 0.02 | 0.02 | 0.03 | 0.03 | 0.03 | 0.04 | 0.06 | 0.06 | 0.07 |
| A1 | 0 | 0.02 | 0.02 | 0.02 | 0.03 | 0.03 | 0.03 | 0.03 | 0.03 | 0.06 | 0.07 | 0.07 |

b)

**Pairwise geographic distance (km)**

| Site | A1 | A2 | A3 | A4 | A5 | A6 | A7 | A8 | A9 | A10 | A11 | A12 |
|---|---|---|---|---|---|---|---|---|---|---|---|---|
| A12 | | | | | | | | | | | | 0 |
| A11 | | | | | | | | | | | 0 | 5 |
| A10 | | | | | | | | | | 0 | 19.9 | 24.6 |
| A9 | | | | | | | | | 0 | 18.6 | 38.3 | 43.1 |
| A8 | | | | | | | | 0 | 12.8 | 28.1 | 46.1 | 51.1 |
| A7 | | | | | | | 0 | 14 | 26.7 | 39.7 | 55.3 | 60.2 |
| A6 | | | | | | 0 | 7.4 | 18.5 | 31.1 | 45.8 | 62.2 | 67.1 |
| A5 | | | | | 0 | 4.6 | 10.3 | 22.8 | 35.5 | 49.8 | 65.6 | 70.5 |
| A4 | | | | 0 | 6.7 | 8 | 15.3 | 25.2 | 37.3 | 53.1 | 70 | 74.9 |
| A3 | | | 0 | 6.8 | 12.7 | 14.7 | 22.1 | 31.5 | 43.3 | 59.6 | 76.7 | 81.6 |
| A2 | | 0 | 9.3 | 14.9 | 18.8 | 22.2 | 29.1 | 40.1 | 52.2 | 67.9 | 84.3 | 89.2 |
| A1 | 0 | 13.3 | 20.5 | 27.1 | 31.8 | 34.8 | 42 | 52 | 63.4 | 80 | 97 | 102 |

## Supplementary Materials

**Figure S2**. Principal Component Analysis of *Astartea leptophylla* populations to identify genetic structure present including a) axis 1 and 2, and b) axis 1 and 3. Populations A10, A11 and A12 are highly differentiation from the other sites in the middle and lower catchment; however only a small amount of variation is explained by the axes.

a)

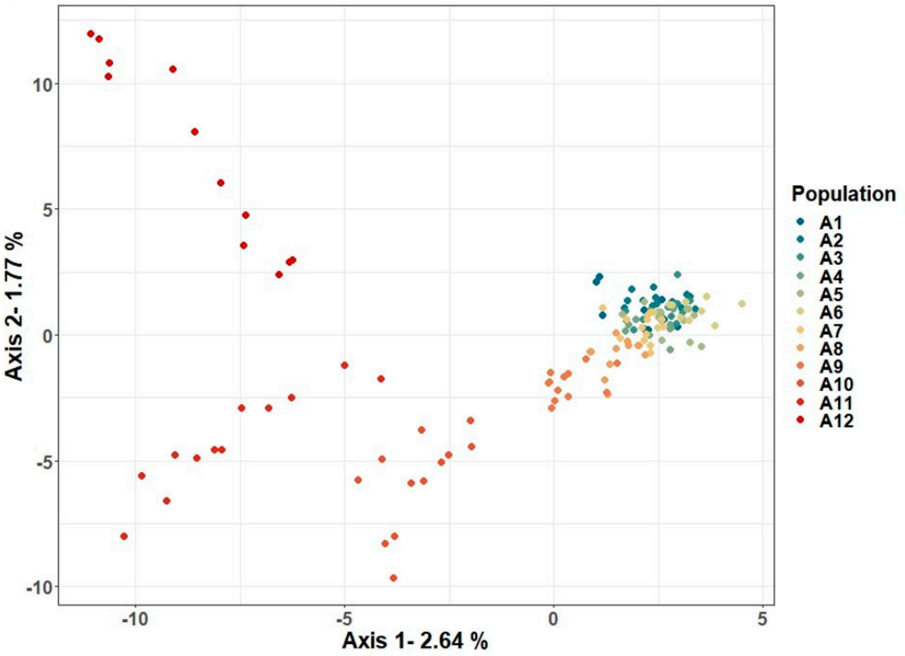


b)

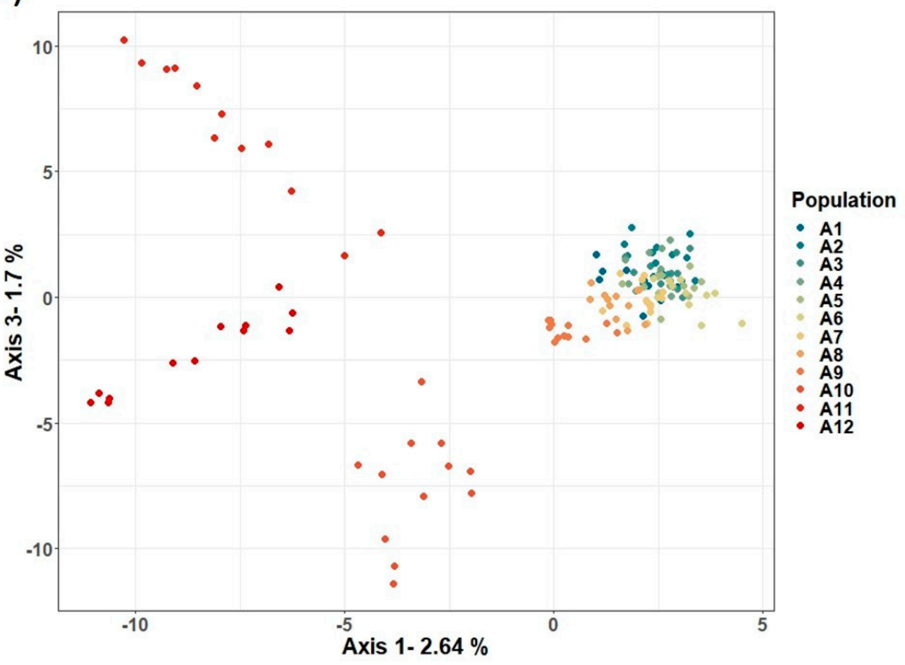

## Supplementary Materials

**Figure S3**. Principal Component Analysis of subset of *Astartea leptophylla* populations, the highly differentiated populations A10, A11 and A12 have been removed to focus on the genetic structure present in the lower and middle catchment, including a) axis 1 and 2, and b) axis 1 and 3. The finer look at these populations identifies populations A1, A2 and A3 grouping together in the negative space of the first axis and populations A8 and A9 grouping in the negative space of the second axis.

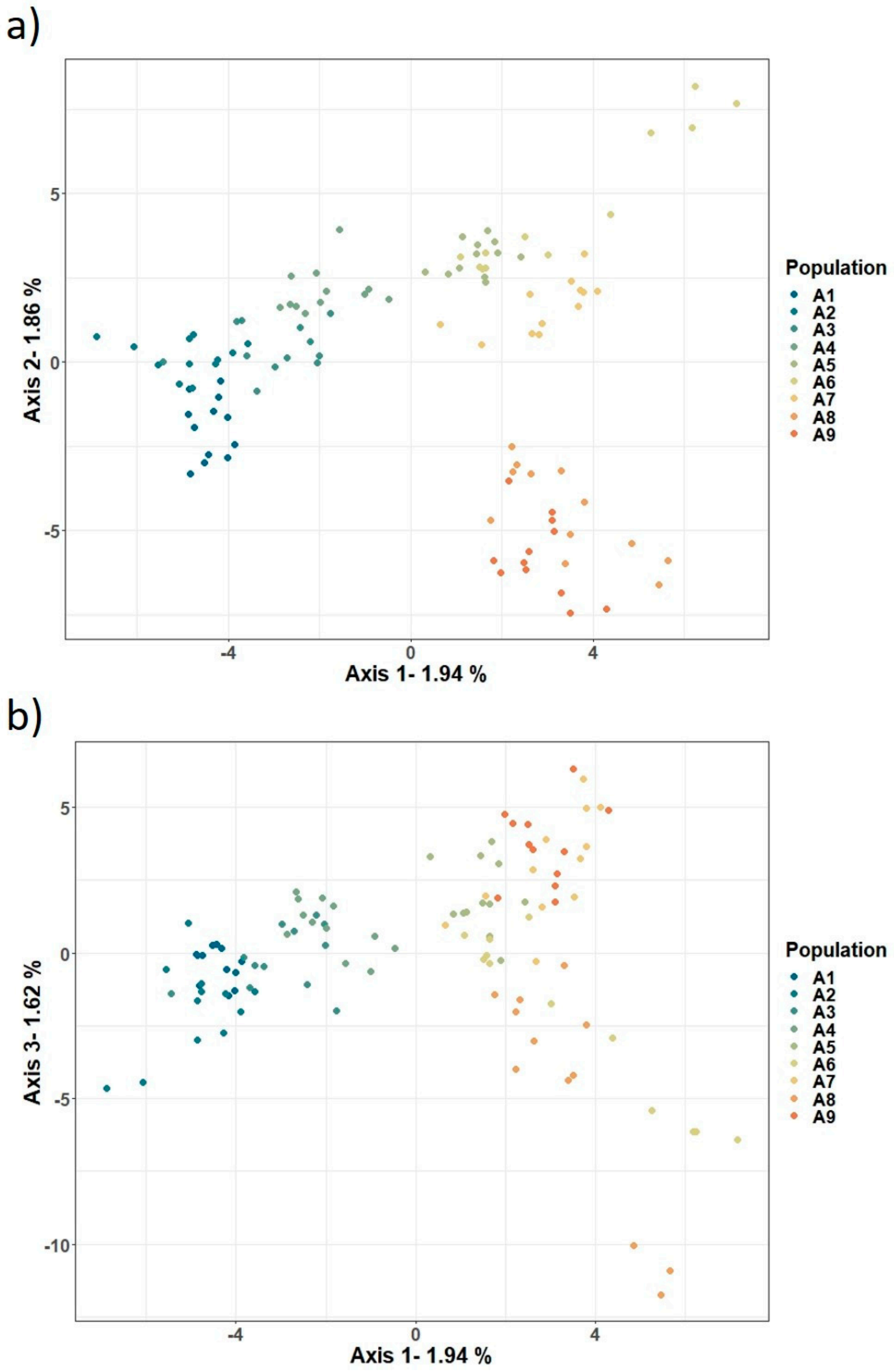

## Supplementary Materials

**Figure S4.** Genetic diversity characteristics for *Astartea leptophylla* populations including, a) number of private alleles, b) expected heterozygosity, c) observed heterozygosity and d) inbreeding coefficient.

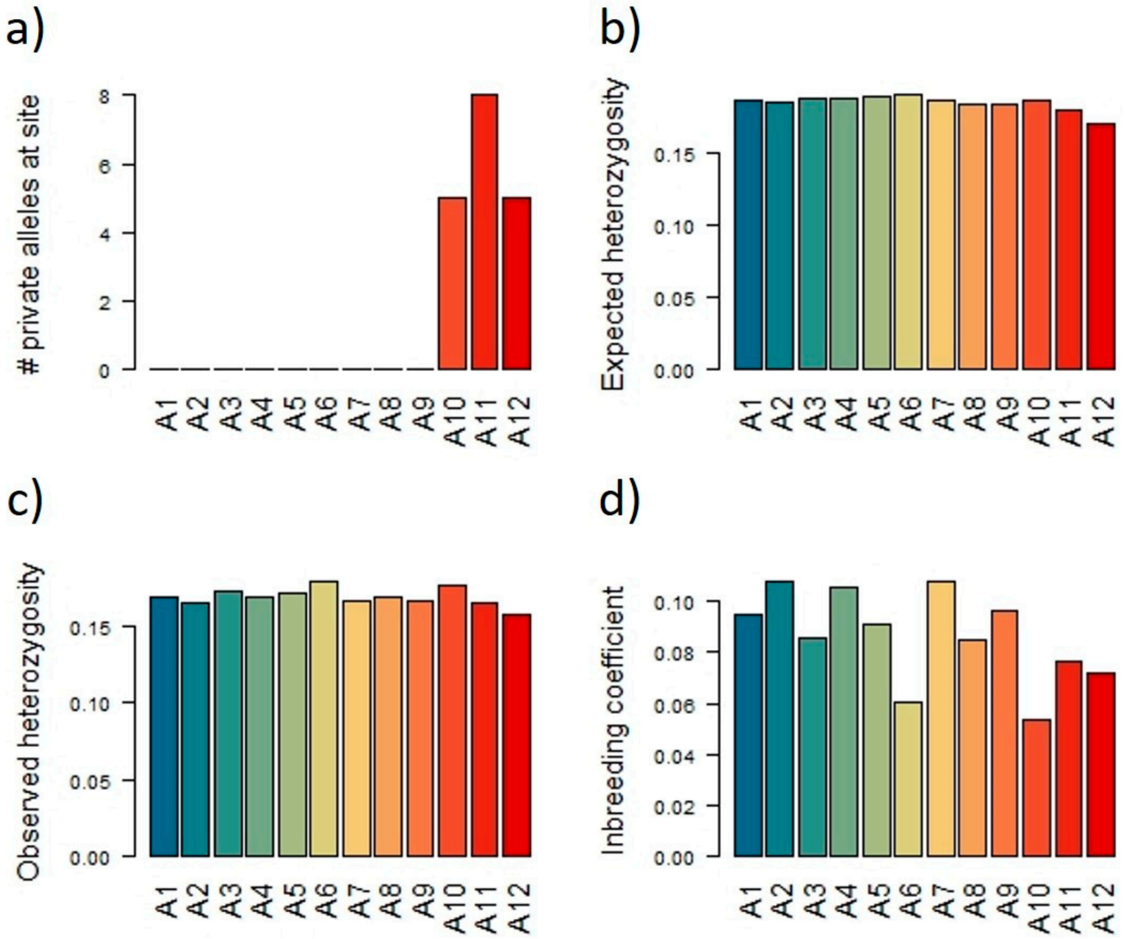

## Supplementary Materials

**Figure S5**. Pairwise a) genetic distance ($F_{ST}$) and b) geographic distance (km) among 12 sites for *Callistachys lanceolata.*

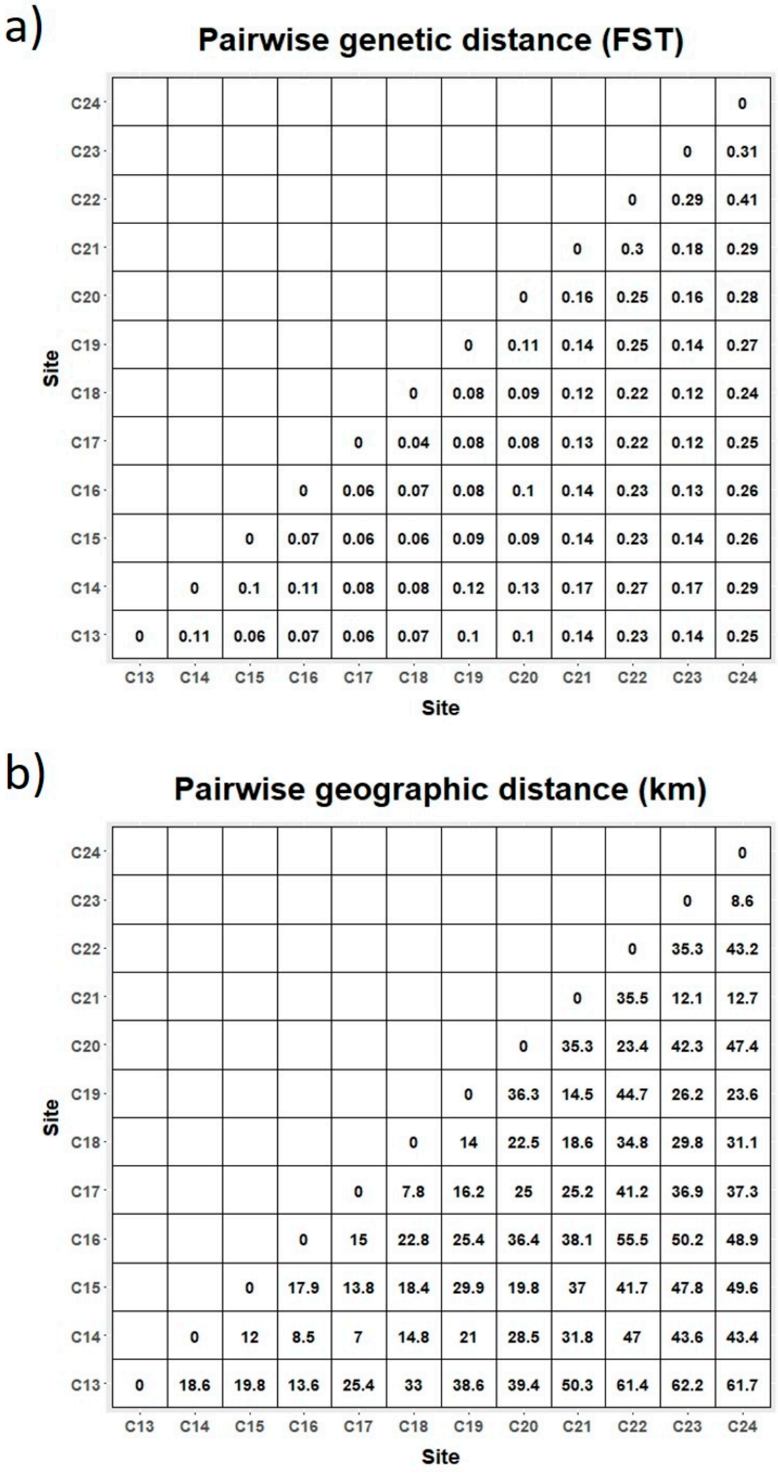

## Supplementary Materials

**Figure S6**. Principal Component Analysis of *Callistachys lanceolata* populations to identify genetic structure present including a) axis 1 and 2, and b) axis 1 and 3. Populations C23 and C24 are highly differentiation in the first and second axes, while populations C21 and C22 are differentiated from the other sites along the third axis.

a)

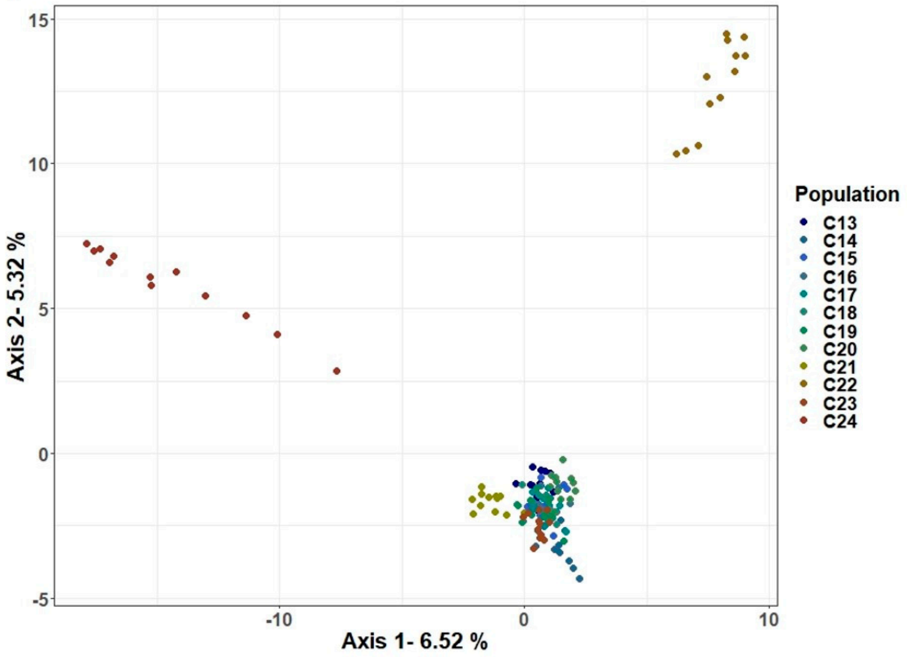


b)

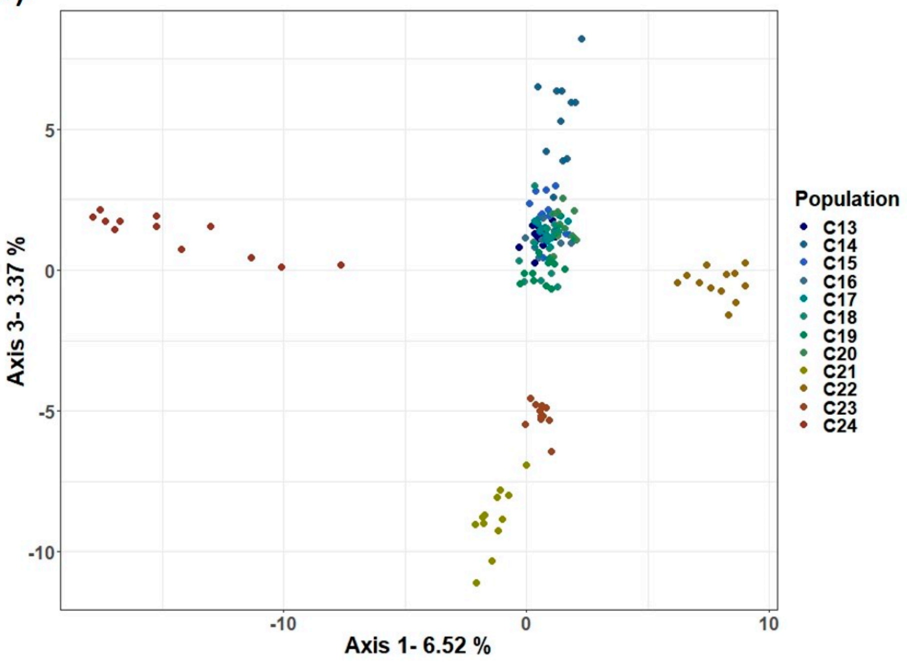

## Supplementary Materials

**Figure S7**. Principal Component Analysis of subset of *Callistachys lanceolata* populations, the highly differentiated populations C21. C22. C23 and C24 have been removed to focus on the genetic structure present in the remaining populations, including a) axis 1 and 2, and b) axis 1 and 3. The finer look at these populations identifies population C14 differentiated on the first axis and populations C19 and C20 grouping together in the positive space of the second axis.

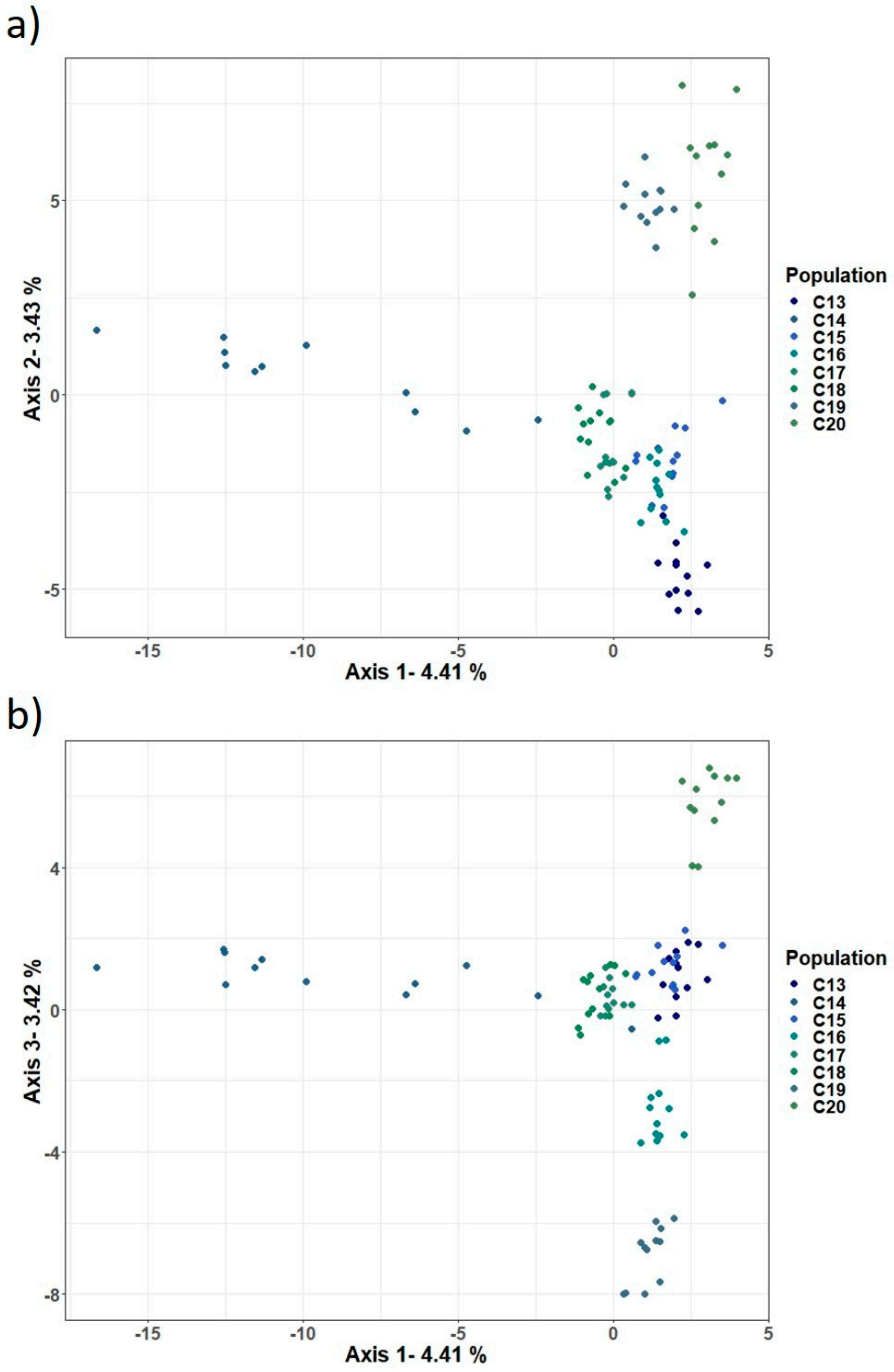

## Supplementary Materials

**Figure S8.** Genetic diversity characteristics for *Callistachys lanceolata* populations including, a) number of private alleles, b) expected heterozygosity, c) observed heterozygosity and d) inbreeding coefficient.

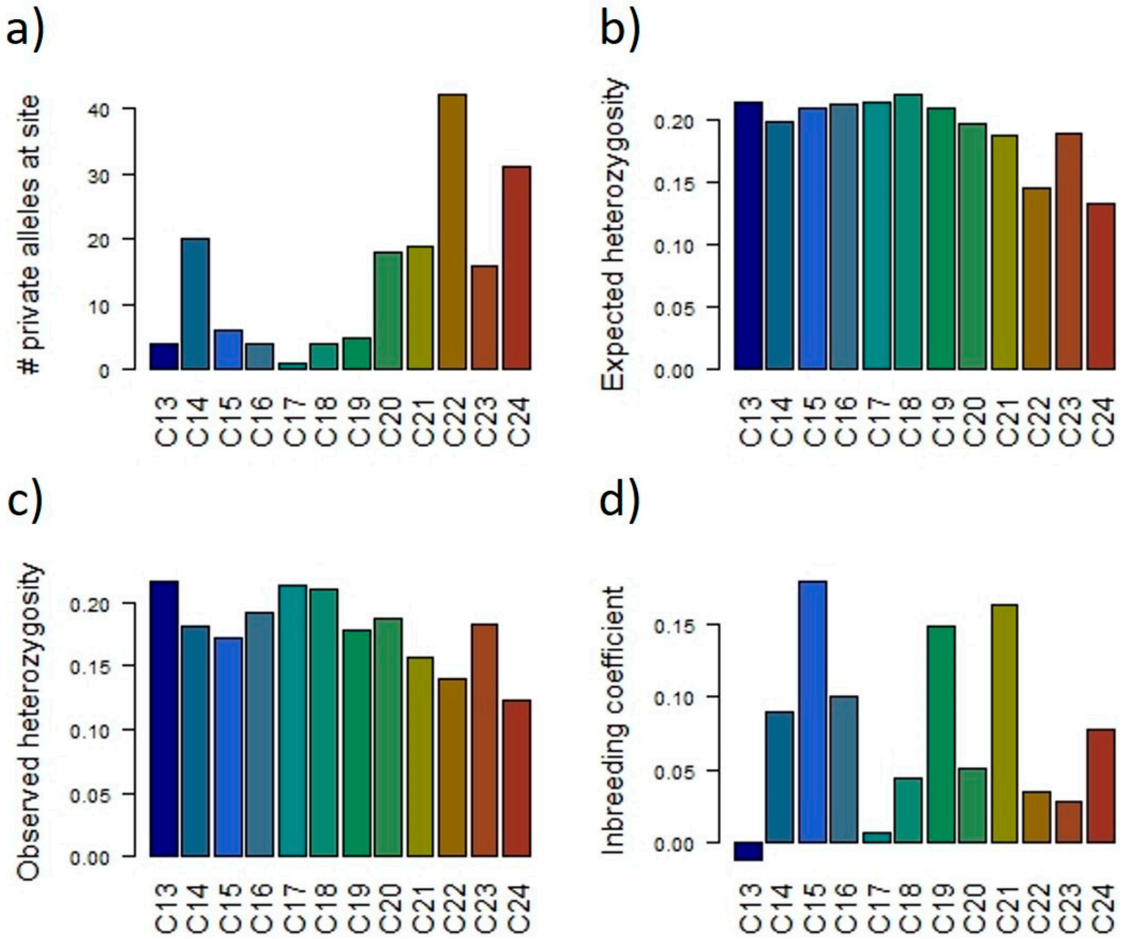

## Supplementary Materials

**Figure S9.** Values for each site of the nine environmental variables tested for associated with loci, standardised value for comparison, for sites of a) *Astartea leptophylla* and b) *Callistachys lanceolata.*

a)

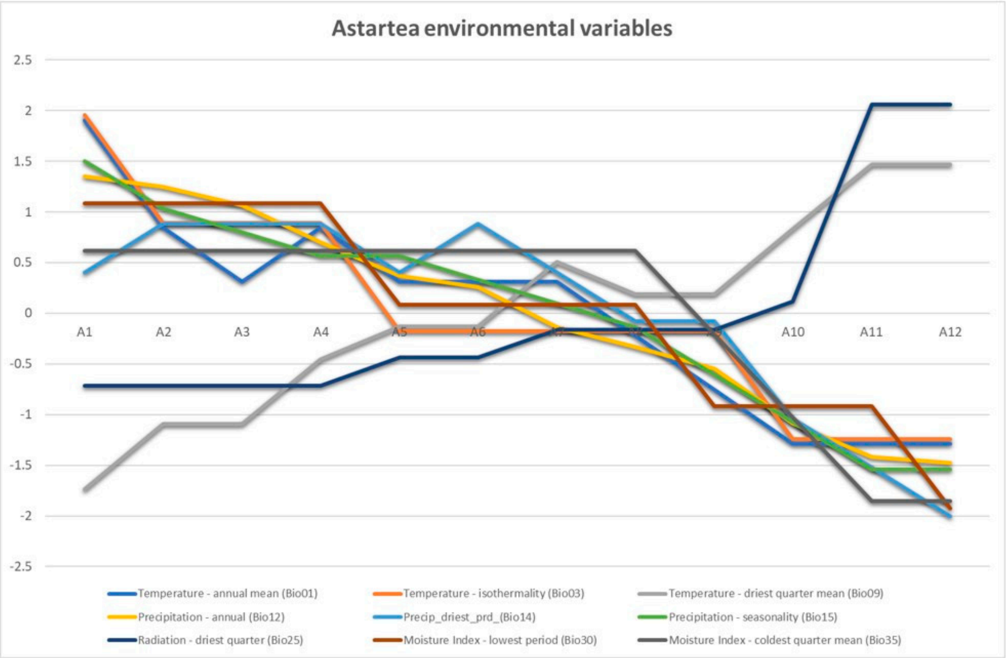


b)

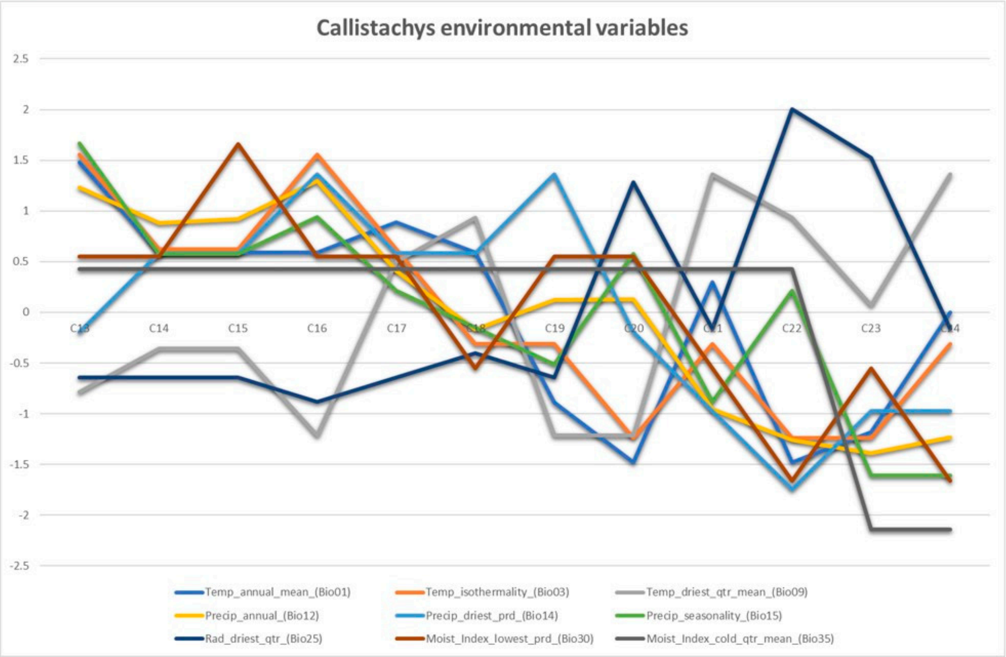

Supplement: Supplementary file 1 [file genes-10-00579-s001.pdf]
